# Management of orthodontic emergencies during 2019-NCOV

**DOI:** 10.1186/s40510-020-00310-y

**Published:** 2020-04-07

**Authors:** Alberto Caprioglio, Giulia B. Pizzetti, Piero Antonio Zecca, Rosamaria Fastuca, Giuliano Maino, Ravindra Nanda

**Affiliations:** 1 Orthodontic Program, Department of Medicine and Surgery, University of Insubria, Varese, Italy; 2 Postgraduate School of Orthodontics, University of Ferrara, Ferrara, Italy; 3 Division of Orthodontics, University of Connecticut Health Center, Farmington, CT USA

The coronavirus (COVID-19) epidemic is a public health worldwide problem for which specific guidelines are published, constantly updated by the World Health Organization (WHO) and, in Italy, by the Italian National Institute of Health. The competent ministries and the regions directly or indirectly contribute to risk management through the identification of suspected cases and the activation of containment and quarantine measures for people who have had contact with suspicious cases.

In the dental field, for the purpose of controlling COVID-19 infection, the fundamental preventive measure lies in the filter of patients who come to the ambulatory. Therefore, a questionnaire should be used to screen patients with potential infection of 2019-nCoV before they could be led to the dental chair-side, as recently suggested [1].

Another fundamental aspect is the correct use of personal protective equipment (PPE) and the strict compliance with the behavioral guidelines at the dental office established by the competent authorities and recently published [1].

Given that the professional can assess whether to stay open or manage emergencies only, common sense must prevail during a quarantine period.

One choice that can be made is to postpone routine orthodontic appointments, according to the guidelines of the single Nations, but patients need to be assured and followed, especially if they are experiencing discomfort or problems related to the orthodontic appliance they are using.

An orthodontic emergency might be described as a problem arising from an orthodontic appliance, where an unscheduled appointment is required to resolve the issue. When a patient has such an issue, a timely additional appointment may need to be arranged with a specialist. Patients who present with an orthodontic emergency may be experiencing pain or discomfort. It can also be inconvenient for the patient and parents in attending for an additional, unexpected appointment due to pre-existing school or work commitments. Consequently, repeated breakages prolong treatment time and can lead to decreased patient motivation due to a loss of confidence in the appliance or the operator. By providing appropriate timely management, inconvenience and distress to both the patient and parents may be minimized with the efficacy of the appliance still being maintained [2].

According to single Nations guidelines during COVID-19 pandemic, dentists should accept in the private practice only non-deferrable urgencies, such as an abscess or irreversible pulpitis. Orthodontic problems, like general dentistry problems, represent urgencies, not true emergencies, so a video call or message with a photo might be the best options to evaluate the case.

A brief summary of guidelines on the management of orthodontic patients during COVID-19 emergency is proposed as follows focusing on virtual assistance devices and classification of emergencies.

## Virtual assistance

WhatsApp Messenger (Facebook Inc., Mountain View, California) is an instant messaging application developed in 2009 and quickly spread among users of all ages, for personal relationships, as entertainment, as an aid to the study, and as a virtual place of contact with their group. The international scientific literature on the use of this application in the health sector, identified by the major database, on-line, reports only a small number of publications. Although its impact in the clinical setting has been poorly investigated, WhatsApp is among the most widely used communication tools, which may also be valuable in favoring the communication and relationship between patients and physicians. Healthcare providers should be trained to use modern web-based communication systems with accurate assessment of indications and contraindications [3, 4].

Clement has recently published some data about the most popular global mobile messenger apps based on the number of monthly active users on Statista (https://www.statista.com/statistics/258749/most-popular-global-mobile-messenger-apps/), which is a German web portal for statistics. The most popular messenger app turns out to be WhatsApp, with 1.6 billion users on a monthly basis.

Nowadays, WhatsApp is the largest messaging app, and therefore, it is the most widespread and most usable even by inexperienced audiences.

The best way to manage orthodontic emergencies is to decide step by step. The first step should always be virtual assistance, and WhatsApp may be considered a good tool to do that. The virtual assistance might be performed by using photos, videos (better if with additional light source), or video call.

In particular, we recommend the business version of WhatsApp to link it to the firm’s fixed number, activating the verification on call.

We recommend doing a triage via WhatsApp to skim the real urgencies to be managed in the private practice in person from remotely manageable situations. It is recommended for large offices or hospital departments to activate WhatsApp Web and have the QR code sent by the dental office staff on duty in order to manage problems on two users.

## Classification of orthodontic emergencies

We can classify orthodontic emergencies on the basis of the type of the appliance used by the patient: removable appliance or fixed appliance.

### Removable appliances

Removable appliances can be classified into the following:

- Functional
- Aligners
- Retainers (for example Essix appliance)

*Functional appliances* are used by growing patients to guide the correct growth of the dento-alveolar complex and the jaws [5].

If the patient breaks the appliance or has noticeable discomfort by wearing it, we suggest suspending the use of the appliance for the moment, in order to reduce emergencies that cannot be managed directly.

An *aligner* or a *retainer* appliance can be often broken or lost by the patient. For aligner treatment, the advice would be to remain on the current aligner if the patient does not have any more until the end of the emergency.

If there are no problems with the current aligner and subsequent aligners are in the patient’s possession, the suggestion is to continue with the subsequent aligner, up to the treatment phase prior to IPR, replacement of attachments, and introduction of elastic modules.

In the event that the current aligner is broken or lost, the advice would be to go on the previous aligner or to change to the next one depending on the percentage of usage of the broken/lost aligner.

As for the *retainers* or if there is a high risk of recurrence that irreversibly compromises the treatment, we suggest to buy an easy contenitive appliance, like hot customizable preforms, which can be found on e-commerce sites such as Amazon, which currently still allows fast shipments, so when the emergency will be finished, the clinician can take new impressions or scans.

### Fixed appliances

Fixed appliances can be classified into the following:

- Non-removable appliances
- Non-removable appliances that can be activated by the patient
- Pre-activated, non-removable appliances

For all emergencies, the patient should send photos or videos to confirm the accident.

The pre-adjusted edgewise appliance (straightwire appliance) is the most commonly used *non-removable appliance* [6]. It includes bands and brackets, archwires, and auxiliaries. The time elapsed since the previous activation is important in order to consider the appliance active or passive; normally, a time of 4 weeks indicates a passive appliance [7].

A bracket may become loose or lose its metallic or elastic ligature as a consequence of eating hard or sticky foods: if the bracket remains flush with the tooth, it can be left as it is, if it seems to fall from the archwire, the patient can carefully try to remove it with eyebrow tweezers.

If there is a metallic ligature that causes soft tissue trauma or pain, the patient should try to push it back with the small eraser on the back of a pencil. In the event that it is not possible, then orthodontic relief wax can be applied. If wax is not in possession of the patient, it might be found in pharmacies or on online stores such as Amazon. In case of an emergency, food wax can be used, since they are both made with micro-crystallized paraffin.

Another very frequent problem, especially during the first phases of the treatment, is protruding distal ends of archwire that can cause soft tissue trauma and large and painful ulcers. If the archwire has slid round to one side, then it may be possible to reposition it with the help of eyebrow tweezers. If the patient is not able to reposition the wire, the best option is to cut it. Thin wires can be cut using a nail clipper. Disinfection can be performed by boiling the instrument in 100 °C water for 30 min [8, 9]. If the wire is thick, it is recommended to try to cut it with a hardware cutter that could be ordered on e-commerce sites without concerns.

In any case of soft tissue trauma caused by sharp objects (end of the wire or ligatures), orthodontic relief wax is a good momentary solution. It can be found in drugstore, pharmacies, and e-commerce sites without problems.

Non-removable appliances that can be activated by the patient are for example the ones that use fixed molar bands but should be worn by the patient, such as face masks, headgears, or lip bumpers.

This type of appliances, and also elastics, should be suspended a priori to reduce the risk of emergencies until the patient can be referred back to the orthodontist.

If the patient feels pain, redness, and swelling near a fixed orthodontic appliance, we can ask him/her to take a photo and send it to the dentist: if a periodontal abscess is suspected, then it is suggested to visit him to remove the cause, for example, a band under the gum, and then to treat the infection with antibiotic therapy. Whether this is not immediately possible, we recommend prescribing a symptomatic therapy with FANS or paracetamol after properly asking for allergies [6, 10].

Fixed treatment can also enclose *pre-activated* appliances, such as Pendulum, Forsus, Distal Jet appliance, and transpalatal bar. In this case, it is recommended to take a picture every 3 weeks and eventually let the patient come to the office to remove it if it represents or could represent an emergency (for example, in the case of pain or swelling).

We must keep in mind that, in this case, the rationale is to prevent emergencies, not to cure them. Moreover, it would be useful to ask the patient to take careful notes on what he did and when. A summary of the possible scenarios and how to solve them is presented in Table 1.

**Table 1:** Orthodontic emergency scenarios and how to resolve them

**Removable appliances**	*Functional*	If it is broken or does not fit, send photos to the orthodontist and suspend the use	
	*Aligners*	Remain on the current/go on with treatment following clinician’s indications/if broken or lost get back to the previous and ask the clinician	
	*Retainers*	If broken or lost, ask to the dentist to evaluate buying hot customable preforms on e-commerce sites	
**Fixed appliances**	*Non-removable appliances (e.g., straightwire appliance)*	Loose bracket	Send a photo to the dentist, eventually remove it with tweezers
		Poking distal wire	Send a photo to the dentist, use wax, eventually cut with disinfected nail clipper/hardware cutter
		Poking ligature	Send a photo to the dentist, use wax, eventually push it back with eraser of a pencil
		Periodontal abscess around molar band	Send a photo to the dentist, symptomatic therapy with FANS/paracetamol, eventually prescription of antibiotic
	*Non-removable appliances activated by the patient* (e.g., face masks, headgears or lip bumpers, palatal expanders)	Must be suspended a priori to avoid future emergencies	
	*Pre-activated, non-removable appliances* (e.g., Pendulum, Forsus, Distal Jet appliance, transpalatal bar)	Take a picture every 20–40 days; if the patient feels pain or swelling, see as an emergency in the dental office and eventually remove the appliance	

## Conclusions

In conclusion, a good method to manage emergencies, reassure, and follow patients remotely, while they are in their home, is via WhatsApp web.

The orthodontist should not let the patient use anything that could generate an urgency in the office such as appliances that can be activated by the patient (elastics, face masks, headgears, lip bumpers, or other non-removable appliances that can be activated by the patient).

At the moment, it is essential to manage in the office with the necessary PPE only the real cases of urgencies that cannot be resolved remotely by the patient, following the guidelines dictated by the WHO and local authorities.
